# Aluminum stress responses and beneficial bacterial traits in *Medicago* legumes

**DOI:** 10.3389/fpls.2026.1801139

**Published:** 2026-04-10

**Authors:** Thomas Ledger, Alexander Renlund, Ángela Cantillo-González, María Josefina Poupin, Bernardo González

**Affiliations:** 1 Laboratorio de Bioingeniería, Facultad de Ingeniería y Ciencias, Universidad Adolfo Ibáñez, Santiago, Chile; 2 Center of Applied Ecology and Sustainability (CAPES), Santiago, Chile

**Keywords:** alfalfa, aluminum, *Medicago*, metals, PGPR, rhizosphere, tolerance

## Abstract

Legumes of the genus *Medicago* are agronomically important forage crops that also enhance soil fertility through biological nitrogen fixation. Beyond their agricultural value, *Medicago* species show promise for the ecological restoration of degraded soils, particularly through their symbiotic associations with soil microbial communities (rhizobacteria). However, in acidic soils—common in degraded environments—the presence of toxic metals such as aluminum (Al) poses a major constraint to plant establishment and microbial functioning. However, the specific impacts of Al stress on each symbiotic partner—and on the dynamics of their interaction—remain poorly understood. This review systematizes and describes recent advances in the effects of Al on *Medicago* legumes, which underlie increased tolerance to metal phytotoxicity, and aims to identify synergistic functions among plant and microbial partners. Al produces morphological and functional changes in *Medicago* species. Key strategies for metal tolerance involve detoxification mechanisms, such as organic acids production, that effectively mitigate the stress caused by metallic ions. Diverse plant growth-promoting rhizobacteria (PGPR) contribute significantly to each of these strategies, either by the direct production of metal-chelating compounds or by the induction of metal sequestration and/or transport functions in the host. These microorganisms, alone or in combination, display traits that can influence Al mobilization and removal for phytoremediation applications. Mechanisms underlying the effect of PGPR on *Medicago* gene expression during metal exposure have begun to be elucidated, as has the role of symbiotic interactions with arbuscular mycorrhizae. Additional studies employing transcriptomics, metabolomics, and genetic engineering are also necessary to fully understand their impact on common metal stress responses and tolerance mechanisms in the genus *Medicago*.

## Introduction

Some metals are naturally present in soils; however, their concentrations can rise due to anthropogenic factors such as changes in soil properties, fertilizer application, and industrial waste disposal (Singh et al., 2016). In acidic soils (pH < 5.5), the availability and assimilation of several cations—including Al³^+^, Cu²^+^, Fe³^+^, Mn²^+^, and Zn²^+^—increase significantly (Rezaeian et al., 2020). While elements such as Co, Cu, Fe, Mg, Mo, Ni, and Zn are essential in small quantities (Tiwari and Lata, 2018), others such as aluminum (Al), arsenic (As), beryllium (Be), cadmium (Cd), chromium (Cr), lead (Pb), mercury (Hg), and selenium (Se) can disrupt plant growth and physiological processes even at low concentrations (Ojuederie and Babalola, 2017; Singh et al., 2016; Tiwari and Lata, 2018). Al is the most abundant metal in the Earth’s crust, mainly present as aluminosilicates and Al oxides (Matsumoto and Motoda, 2012). Its damaging effects are intensified in tropical and subtropical regions, where acidification occurs rapidly due to nutrient cycling, excessive fertilizer use, and pollution leading to acid rain (Rahman et al., 2018).

The chemical composition of soils—including pH, mineral content, and organic matter—strongly influences metal speciation and plant uptake. Once metallic ions reach plant tissues, they are sensed by membrane protein receptors or ion channels, initiating signaling cascades involving ROS production, MAPK activation, and Ca²^+^-dependent processes (Tiwari and Lata, 2018; Yang et al., 2019). Although ROS serve essential signaling roles in regulating stress responses and normal growth (Pinedo et al., 2015), excessive ROS accumulation under metal stress leads to oxidative damage, including macromolecular oxidation, protein misfolding, disrupted hormone pathways, membrane destabilization, and potential programmed cell death (Huang et al., 2019; Tiwari and Lata, 2018). Phytohormones coordinate many of these responses by regulating metal detoxification pathways, activating metal transporter genes, and inducing the synthesis of chelators that maintain cellular homeostasis (Li et al., 2014; Tiwari and Lata, 2018; Yang et al., 2019; Chakraborty et al, 2024).

Legumes (*Fabaceae*) play a pivotal role in improving soil characteristics and enhancing fertility by replenishing nitrogen (N) through symbiosis with rhizobacteria (Adams et al., 2018). These plant–microbe interactions offer additional ecosystem benefits, including enhanced soil fertility (de Lima Coêlho et al., 2024), support for other plants and microorganisms (Bouchenak and Lamri-Senhadji, 2013), improved soil structure (Zhao et al., 2014), and reduced soil metal levels (Raklami et al., 2019). Among legumes, *Medicago sativa* (alfalfa or lucerne) stands out for its broad agroecological impact and versatile use in forage production, soil regeneration, and phytoremediation (Zhou et al., 2024). It is a high-yielding perennial crop in temperate climates and a source of green manure, bioactive compounds, and nutrient-rich food products (Duarte et al., 2024; Rezaeian et al., 2020). The closely related annual species *M. truncatula* has become a premier model legume due to its compact genome (500–550 Mbp), facilitating studies on rhizobial interactions, arbuscular mycorrhizal fungi (AMF) associations, root architecture, and secondary metabolite biosynthesis (Gholami et al., 2014). Similarly, *M. lupulina* has been studied for its symbioses with *Sinorhizobium* spp. and AMF, particularly under heavy-metal stress (Chou et al., 2019). Symbiotic associations between *Medicago* species and soil microbes—particularly rhizobacteria and AMF—are central to nutrient cycling, soil formation, and plant tolerance to stress. While Medicago species are not classified as metal hyperaccumulators, their rapid growth, high biomass, expansive root systems, and tendency to retain metals in roots make them promising for grazing-compatible phytoremediation strategies (Raklami et al., 2019). Understanding how metal stress influences these plant–microbe partnerships is essential for enhancing both plant resilience and soil restoration.

This review integrates current knowledge on the physiological and molecular responses of *Medicago* to metal stress, particularly Al; the role of microbial partners (rhizobacteria, AMF, and others) in metal tolerance; and how plant–microbe synergistic mechanisms can be leveraged to enhance growth and resilience in metal-impacted environments. Such an integrated perspective is crucial given the need to rehabilitate degraded soils and develop sustainable legume-centered agricultural systems (Chen et al., 2024; Ojuederie and Babalola, 2017). Despite substantial progress, several gaps persist, including an incomplete understanding of the molecular mechanisms of Al tolerance in *Medicago*, limited information on how soil chemistry modulates symbiosis under metal stress, insufficient characterization of microbial taxa that enhance metal tolerance, and challenges in translating lab research into field-scale phytoremediation and sustainable agriculture. Addressing these gaps will improve our ability to design effective plant–microbe systems to restore metal-contaminated soils and enhance legume performance.

## Effects of metal stress on *Medicago* plants

### Growth and developmental effects

The primary effect of metal stress in *Medicago* legumes, as with other plant species (Chakraborty et al, 2024), is a significant reduction in shoot and root growth and development, revealed upon exposure and accumulation of Al, Cr, Cd, Cu, Ni, Pb, and Zn, as reported for *M. lupulina*, *M. sativa*, and *M. truncatula* (Chou et al., 2019; Cui et al., 2013; Peralta-Videa et al., 2002; Sun et al., 2007; Wang et al., 2017; Yahaghi et al., 2019). Concerning *M. sativa*, significant inhibition of root growth occurs upon exposure to Al/acidity *in vitro*, even at micromolar concentrations (Cui et al., 2013, 2017; Su et al., 2020; Tesfaye et al., 2001; Zhou et al., 2014). A similar effect is observed in phytoremediation studies, in which *M. sativa* shoot and root biomass is considerably reduced following exposure to Cd^2+^, Cu^2+^, Ni^2+^, Pb^2+^, and Zn^2+,^ alone or in combination, at pH 4.5 (Peralta-Videa et al., 2002). In general terms, phytotoxicity levels are high for Pb^2+^, Ni^2+^, Cu^2+^, and Al^3+,^ and even higher for Cd^2+^ and Cr^6+^, which exhibit the most severe effects on *Medicago* elongation and dry weight (Das et al., 2021).

Another relevant indicator of metal stress in *Medicago* species is seedling germination, which is impaired due to exposure to Al, Cd, Cr, Cu, Pb, and Zn (Raklami et al., 2021; Samma et al., 2017; Yahaghi et al., 2019; Zhou et al., 2016). Under slightly acidic conditions, a sharp decline in alfalfa germination with concentrations above 6.4 mM Al^3+^ is observed (Zhou et al., 2016), while a Cu concentration of 1.5 mM reduces the germination rate down to 54.9% (Samma et al., 2017), which is a consistent effect among different varieties of *M. sativa* (Raklami et al., 2021). The detrimental impact on seed germination is ascribed to interference with the catalytic activity of various metalloenzymes required for macronutrient hydrolysis, embryo development, and further seedling metabolism (Raklami et al., 2021; Yahaghi et al., 2019).

**Figure 1:**
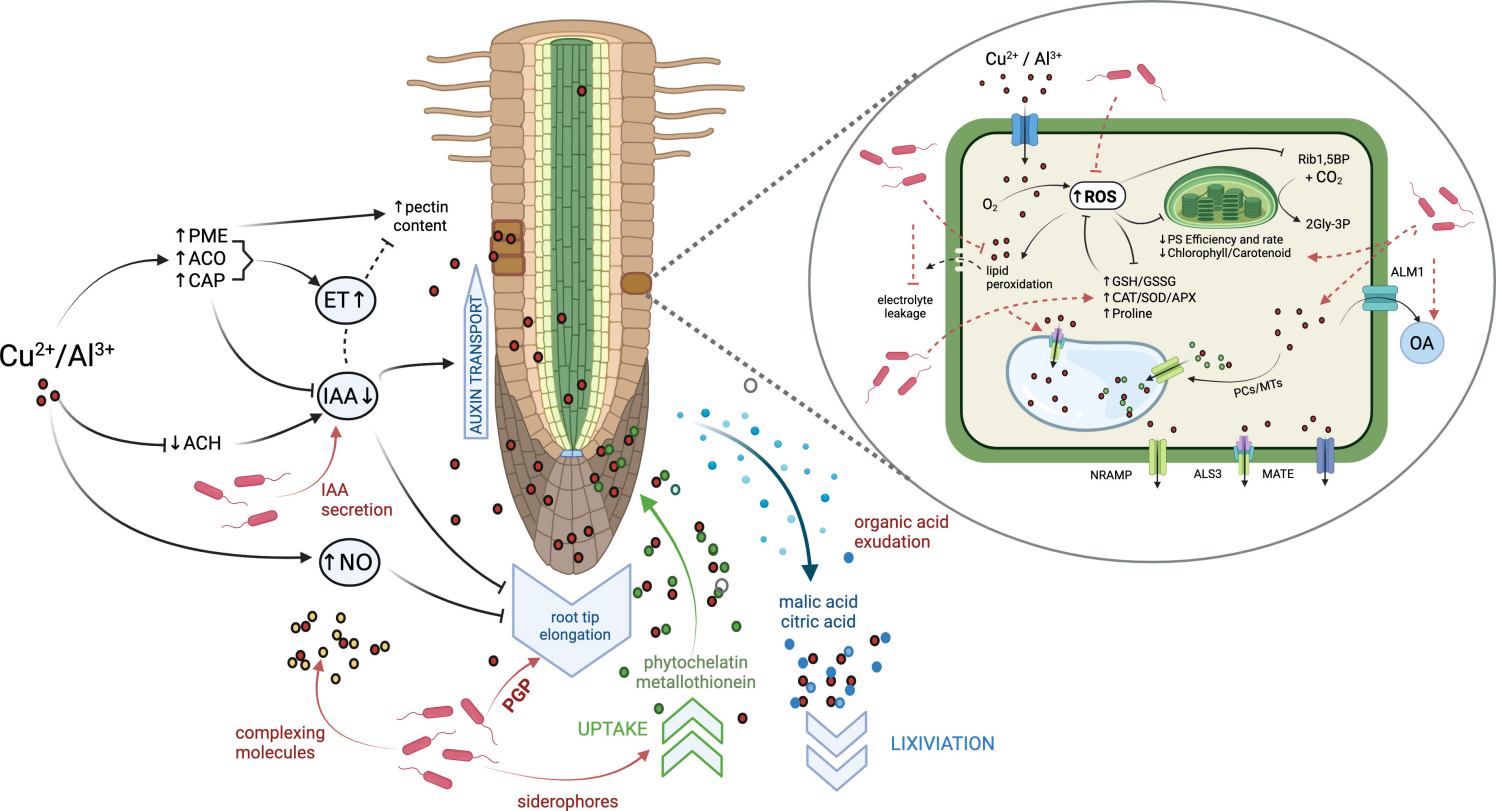
Interactions of *Medicago* plants with metals and rhizobacteria at the root tissue level. Metals, including Cu^2+^ and Al^3+^, but not limited to them, have been shown to inhibit root growth by altering phytohormone balance in the root meristematic tissue. Specifically, metals have been shown to reduce auxin (IAA) and increase ethylene (ET) content by modulating different metabolic pathways (left), such as inducing CAP peroxidase for IAA degradation and further reducing auxin content by increasing IAA transport from the meristem to the upper root zones; inducing pectin methylesterase (PME) elevating pectin content and 1-aminocyclopropane-1-carboxylase (ACO) increasing ET levels. Nitric oxide (NO) production has been shown to contribute to root growth inhibition. Rhizobacteria, including those that promote plant growth (middle); secreting IAA (left), chelating and immobilizing metals at the rhizoplane or by facilitating root uptake, through siderophores, phytochelatin and metallothionein (PCs and MTs), release of non-toxic complexes (bottom); by producing organic acids and/or stimulating root organic acid (OA) production (i. e. citric and malic acids), which increases metal solubility and lixiviation from the root zone (right). Inset: Additional effects of rhizobacteria include increased production of reactive oxygen species (ROS) and increased expression of organic acid transporters. When Cu^2+^, Al^3+^, and other ions enter plant cells via ionic channels or membrane transporters, they alter oxygen metabolism, leading to ROS accumulation, lipid peroxidation, and damage to the cytoplasmic membrane, resulting in electrolyte leakage (left). ROS also alter chloroplast structure, reducing photosynthetic (PS) rate and efficiency, ribulose-1,5-bisphosphate (Rib1,5BP) synthesis, and CO_2_ fixation, as measured by glyceraldehyde-3-phosphate (Gly-3P) production (right). Rhizobacteria reduce ROS-mediated cell damage by stimulating the production of ROS-scavengers and inducing the expression of diverse metal-complex-transporting proteins, including NRAMPs, metal ion transporters, MATE, and the ABC transporter ALS3, thereby increasing metal efflux to the apoplast and metal-complex accumulation in the cell vacuole. Created with BioRender.com.

Exposure of *Medicago* species to Al³^+^, Cd²^+^, or Cu²^+^ induces notable structural disturbances, including root cell wall thickening and alterations in vacuoles and chloroplasts (Chou et al., 2019; Lv et al., 2023). In *M. sativa* and *M. truncatula*, Al exposure triggers extensive remodeling of cell wall polysaccharides—particularly pectin and hemicellulose—together with lignin deposition, callose accumulation, increased PME activity, and the upregulation of pectin and acetyl esterase genes (**Figure 1**) (Chandran et al., 2008; Wang et al., 2017; Zhou et al., 2016). Although such modifications are often interpreted as defensive mechanisms that enhance Al immobilization, evidence suggests that their functional role is more sophisticated. In alfalfa, the tolerant genotype WL-;550 sequesters Al mainly in pectin, whereas the sensitive WL-;440 accumulates significantly more Al³^+^ in the cell wall and hemicellulose-;1 fraction and shows greater Al entry into the symplast and apoplast, as well as enhanced Al transport to shoots (Wang et al., 2017). Both genotypes exhibit increased pectin methyl esterase (PME) activity, sugar content, hemicellulose levels, and thickened meristematic zones under Al stress, indicating that cell wall remodeling alone does not differentiate tolerance from sensitivity. Instead, these findings highlight that while cell wall Al accumulation contributes to detoxification, it is insufficient as a standalone mechanism. Effective Al tolerance in *Medicago* likely requires coordinated strategies that limit symplastic entry, restrict long-distance transport, and maintain cell wall functionality.

### Phyto-hormone-related signaling in response to metal stress

Plant hormones are central regulators of growth and stress adaptation, and accumulating evidence shows that aluminum (Al³^+^) and other metal toxicities disrupt the biosynthesis, signaling, and distribution of key phytohormones in *Medicago*. Transcriptomic studies consistently associate Al stress with altered expression of genes in auxin (IAA) and ethylene (ET) pathways (Sun et al., 2007; Wang et al., 2016; Chakraborty et al., 2024; Zhang et al., 2024). In *M. truncatula*, Al³^+^ exposure suppresses root elongation while inducing 1-aminocyclopropane-1-carboxylic synthase and 1-aminocyclopropane-1-carboxylic oxidase (ACO) enzymes, which are essential for ET biosynthesis, linking ethylene overproduction to growth inhibition (Sun et al., 2007). However, the sensitive cultivar Jemalong 17 downregulates ACO within 12 h of exposure, suggesting that reduced ET synthesis may be an adaptive rather than a maladaptive response (Chandran et al., 2008). Evidence from *M. lupulina* further confirms metal-induced ET pathway plasticity, with Cu²^+^ activating multiple *ACO* genes and ET-;response regulators (EIN3/EIL1) (Chou et al., 2019), while recent systematic annotation of ACS/ACO families in legumes underscores the complexity underlying ET-related stress responses (Gómez-Fernández et al., 2025). **Figure 1**.

Auxin metabolism is similarly altered during metal stress. In *M. sativa*, Al³^+^ accumulation in the apoplast and cell wall disrupts meristem organization and reduces IAA content (Wang et al., 2016; Zhou et al., 2014), with sensitive cultivars experiencing greater declines than tolerant ones (Wang et al., 2017). Al downregulates the *ACH* gene (involved in producing active IAA) and upregulates CAP (an IAA-oxidizing peroxidase), thereby reducing free auxin and impairing root elongation (Zhou et al., 2014). Transcriptomic analyses demonstrate downregulation of SAUR and AUX/IAA and upregulation of ARFs and ERFs, highlighting extensive auxin–ethylene crosstalk under Al stress (Liu et al., 2017). New findings from somatic embryogenesis research show that phytohormone regulation in *Medicago* involves highly dynamic interactions among auxin, gibberellins, jasmonates, and ethylene, reflecting broad hormonal plasticity under stress (Kępczyńska and Kępczyński, 2023). Other hormones also contribute to metal tolerance. Jasmonic acid (JA) and salicylic acid (SA) improve photosynthesis and root and leaf growth in Al³^+^-; and Cu²^+^-;treated *M. sativa* (Su et al., 2020). Ethylene signaling, now understood to be modulated by respiration and epigenetic mechanisms, requires reinterpretation within the context of metal-;induced stress (Wang et al., 2025). Microbial partners can additionally reprogram auxin distribution and ion homeostasis, enhancing Al tolerance (Zhang et al., 2025). Exogenous IAA application restores H^+^-;ATPase activity and corrects auxin gradients, reversing Al-;induced deformation in *M. sativa* roots (Wang et al., 2017, 2016).

Despite considerable progress, addressing some knowledge gaps will be essential for developing a complete framework of hormone-mediated metal tolerance in *Medicago*. For instance, the hormonal interactions governing stress adaptation under multi-;metal exposure are largely unexplored, even though natural soils rarely contain a single toxic metal; the upstream regulators responsible for genotype-;specific hormonal behavior—particularly why some cultivars suppress ET or maintain IAA levels under stress—remain undefined, and the spatial regulation of auxin and ethylene transporters under Al stress, especially how altered cell wall structure redirects hormone fluxes, is poorly understood.

### Organic acid-mediated metal exclusion efflux transporters

Two well-known systems have been described to explain root stress tolerance under metal exposure. First, an uptake restriction strategy, consisting of external exclusion of metallic ions in the apoplast or root exterior. This is achieved by either adhering ions to the cell wall or secreting low-molecular-weight chelating compounds from root tips (Tesfaye et al., 2001). These actions limit interactions with the plasma membrane, thereby restricting metal uptake and accumulation (Yang et al., 2019). One of the most studied mechanisms in Al tolerance described in various plant species is the exclusion of Al^3+^ ions by organic acid exudation, including malate, citrate, and/or oxalate which allow metal lixiviation (Fan et al., 2024; Rahman et al., 2018) and flavonoids, which also eliminate ROS content caused by Al (Jin et al., 2024). **Figure 1**. The second strategy involves internal detoxification of metallic ions. This is achieved by increasing the synthesis of compounds such as phytochelatins, organic acids, and metallothionein-metal chelators (Mathur and Panwar, 2024). These compounds form non-phytotoxic complexes in the cytoplasm, which can be further sequestered into vacuoles by tonoplast-specific transporters (Jonak et al., 2004; Li et al., 2014) (**Figure 1**).

Even though micropropagation and cultivar selection techniques have allowed the development of vigorous Al^3+^/acid tolerant alfalfa varieties (Kamp-Glass et al., 1993), the mechanisms of Al tolerance have been better understood through genetic mapping of quantitative trait loci and the creation of transgenic plants (Cao et al., 2024; Jin et al., 2024; Zhang et al., 2024) as well as the transcriptional studies (Jin et al., 2025). This research has facilitated the identification of genes associated with Al^3+^ tolerance in *M. sativa*, including malate dehydrogenase and phosphoenolpyruvate carboxylase, which encode enzymes involved in malate synthesis (Tesfaye et al., 2001). They reported that the nodule isoform of the malate dehydrogenase gene, overexpressed in transgenic *M. sativa*, plays a role in Al^3+^ tolerance by promoting the biosynthesis of various organic acids and their exudation from root tips. Other organic acid biosynthesis and exudation effectors have been identified by comparing the phenotypic and transcriptional responses of sensitive and tolerant cultivars of *M. sativa* and *M. truncatula* to similar levels of Al^3+^, Cd^2+,^and Pb^2+^ exposure (Cui et al., 2017; Wang et al., 2017). Transcription factors from the WRKY family have been correlated to cell wall lignin and flavonoid biosynthesis in Al-tolerant *M. sativa* seedling roots under Al^3+^/acid stress (Zhou et al., 2016), and regulatory pathways of STOP1 and WRKY transcription factors could be related to tolerance mechanisms of organic acid secretion, synthesis, and Al^3+^ detoxification in different plant species (Fan et al., 2024; Yang et al., 2019).

As in the case of sequestering proteins, metal stress has been observed to induce the expression of active ionic metal efflux transporters in *M. sativa* and *M. truncatula.* Transcriptomic studies of *M. sativa* roots showed the upregulation of eight putative major facilitator superfamily protein transporters previously reported to sequester Al^3+^ chelated complexes in root cell vacuoles (Liu et al., 2017). Up-regulation of an ABC transporter in *M. truncatula* root tips and mature regions exposed to Al^3+^ has been observed, suggesting its role in detoxifying cells by sequestering Al^3+^ into vacuoles in both tissues (Chandran et al., 2008). Transcriptomic studies indicate that coordinated expression of ion-sequestering proteins and metal efflux transporters is key to detoxification in *Medicago* cells and tissues. Consistently, when examining *Medicago* metal tolerance strategies, a global response that combines organic acid-mediated root exclusion and intracellular chelation–sequestration mechanisms is likely required to counteract metal stress effectively (Jin et al., 2024; Min et al., 2019; Sun et al, 2020).

### Antioxidant response to metal within plant cells

Multiple studies have shown increases in the ROS contents in the cells of roots, shoots, and leaves of *M. sativa* upon exposure to Al^3+^, Cd^2+^, Cr^6+^, and Cu^2+^, which have also been linked to several biochemical, metabolic, morphological, and physiological adverse effects (Cui et al., 2017; Das et al., 2021; Rahman et al., 2014; Su et al., 2020; Tirry et al., 2021). Metal-induced accumulation of ROS, such as •OH, H_2_O_2_, and O_2_^-^, can occur through various mechanisms (Huang et al., 2019). For redox-active metals such as Cu^2+^ and Fe^3+^, this happens via direct redox reactions. In contrast, redox-inactive metals like Al, Cd, Pb, and Hg increase ROS levels by activating NADPH oxidases, displacing essential cations from enzyme binding sites, and inhibiting enzymatic function due to their affinity for –SH groups (Shahid et al., 2014). Excessive ROS synthesis in leaves, following exposure and accumulation of metals, has been implicated in reductions of photosynthetic parameters, such as photosystem efficiency, relative photosynthetic rate, and photosynthetic electron flux, and a reduction in photosynthetic pigment contents, such as carotenoids and chlorophyll II (Das et al., 2021) (**Figure 1**, inset). From a metabolic perspective, Al^3+^ exposure in *M. sativa* impacts primary metabolism by reducing the foliar activity of ribulose-1,5-bisphosphate carboxylase/oxygenase and carbonic anhydrase, respectively, involved in CO_2_ fixation (Cui et al., 2017; Rahman et al., 2014; Su et al., 2020). Additionally, excessive ROS accumulation and subsequent oxidative stress lead to structural damage of cells, particularly membrane lipid peroxidation, in roots and leaves of *M. sativa* upon exposure to Al^3+^, Cd^2+^, and Cu^2+^ (Cui et al., 2017). Lipid peroxidation of the cytoplasmic membrane disrupts its structure and functioning, leading to increased electrolyte leakage and potential reductions in internal osmolarity, which have been reported in *M. sativa* roots, shoots, and leaves due to Al^3+^, Cd^2+^, Cr^6+,^ and Pb^2+^ stress (Das et al., 2021; Tirry et al., 2021) (**Figure 1**, inset).

Reduction of intracellular structural and functional damage caused by ROS molecules involves the synthesis of enzymatic antioxidants, such as ascorbate peroxidase (APX), catalases (CAT), peroxide dismutase (POD), and superoxide dismutase (SOD), or non-enzymatic scavenging amino acids and peptides (Huang et al., 2019; Tiwari and Lata, 2018). For instance, a reduction in antioxidant enzymatic activities APX, CAT, and SOD, and the corresponding diminished gene expression, has been identified in roots and leaves of *M. sativa* due to Al^3+^, Cd^2+^, and Cu^2+^ stress (Cui et al., 2017). At the same time, increases in CAT, POD, and SOD activities in roots and shoots of *M. sativa* have been observed under Al^3+^, Cd^2+^, and Cu^2+^ exposure, although these responses were evidenced along with phytotoxic damaging effects associated with oxidative stress (Cui et al., 2017; Das et al., 2021; Samma et al., 2017). However, Cui et al. (2013) determined a significant genetic upregulation and activity of heme oxygenase 1 enzyme in *M. sativa* roots due to 10 µM Al^3+^ exposure, along with increases in APX, CAT, and SOD, but comparatively lower antioxidant activity under 100 µM Al^3+^, indicating decaying oxidative response mechanisms under higher metal concentrations. This suggests that although antioxidant enzymes are relevant to alfalfa metal tolerance, they are often inhibited or downregulated at high metal concentrations. Other antioxidant properties, including critical amino acids and small molecules such as soluble sugars and proline, increase in alfalfa leaves when exposed to Al^3+^ and Cr^6+^ (Rahman et al., 2014; Tirry et al., 2021). The maintenance of reduced glutathione levels under metal stress, mediated by glutathione reductase activity, has also been reported as a response to Al phytotoxicity (Cui et al., 2017). Regarding the synthesis of other scavenging molecules, elevated activity of glutamine and glutamate synthases in the leaves of *M. sativa* has also been observed under Al^3+^ and Cd^2+^ exposure, indicating activation of the non-enzymatic antioxidant system (Rahman et al., 2014). These observations highlight that metal exposure in M. sativa induces ROS accumulation in various tissues, leading to oxidative stress that impairs photosynthesis, disrupts membrane integrity, and alters metabolic function. While antioxidant enzymes (SOD, CAT, APX, POD) and non-enzymatic compounds such as proline, glutathione (GSH/GSSG), and soluble sugars contribute to ROS detoxification, their effectiveness diminishes at higher metal concentrations (Ansari et al., 2024; Sharma et al., 2025). **Figure 1**, inset. This dose-dependent decline suggests a threshold beyond which antioxidant responses are insufficient to prevent cellular damage, particularly in sensitive *M. sativa* cultivars.

## Microorganisms protecting *Medicago* from metal stress

### Interactions among *Medicago*-PGPR-metals

The rhizosphere microenvironment is a dynamic, molecularly and biologically active soil space influenced by roots (Grover et al., 2021). Root exudates attract beneficial microbes and regulate the growth of detrimental ones (Upadhyay et al., 2022). Root exudation, rhizosphere community structure, and the biochemical activities of plants and microorganisms are strongly influenced by soil physicochemical properties, nutrient availability, and exposure to contaminants, including metal ions (Badri and Vivanco, 2009; Chakraborty et al, 2024). Several studies have examined molecular interactions between *Medicago* legume roots and their neighboring microbial communities to understand rhizosphere dynamics under metal stress, with particular attention to PGPR (this sub-section) and AMF (the following sub-section). Instead, the role of soil protists in *Medicago*-rhizosphere interactions is just beginning to be unveiled (Hawxhurst et al., 2023).

Although natural soil acidification can improve nutrient cycling and solubilization, it can also increase levels of toxic metallic ions. This may reduce the availability of essential nutrients, such as P and N, and alter soil microbial community structure (Saha et al., 2021). Several reports have explored interactions among *Medicago*, rhizosphere microorganisms, and metals (Nonnoi et al., 2012). Duan et al. (2022) observed that excessive multi-heavy metal stress was associated with high metabolic and enzymatic activities related to C, N, and P acquisition, along with higher soil organic C, total N, and total P in the rhizosphere and bulk soil of *M. sativa*. This suggests that associations with *M. sativa* enable the formation of a stable, metabolically efficient microbial community. In addition, *the M. sativa* rhizosphere microbial composition is more diverse under oil and heavy-metal (Al, Ag, Cu, and Pb) pollution than that of *Festuca arundinacea* and *Salix miyabeana* (Brereton et al., 2020), suggesting greater resilience to environmental stressors.

Detrimental effects of Al, Cd, and Cu in the development of root and symbiotic structures, such as nodules, have been shown in *M. lupulina*, *M. sativa*, and *M. truncatula*, altering their viability, morphology, and cell structure (Chou et al., 2019; Das et al., 2021; Wang et al., 2016, 2017). Exposure to heavy metals also reduces nodule formation and effective host colonization in Medicago species, in turn reducing nitrogenase activity and legume root N acquisition (Chou et al., 2019; Guefrachi et al., 2013). Guefrachi et al. (2013) also reported a reduction in nodulation and symbiotic interaction of *M. marina*, *M. minima*, and *M. truncatula* with several Cd^2+^ tolerant or sensitive *S. meliloti* strains under Cd^2+^ exposure. However, co-inoculation of *M. sativa* with *Pseudomonas* sp. strains enhanced *in vitro* nodulation by *Ensifer* sp. rhizobia by almost 100% under As exposure (Flores-Duarte et al., 2022a), while co-inoculation with *Variovorax paradoxus* and *V. gossipyi*, which increased nodulation through *E. medicae* under the same stress (Flores-Duarte et al., 2022b). The nodulation of *S. meliloti* exposed to soil acidity and Al^3+^ also varied among different strains, with superior nodulation in more tolerant varieties (Wigley et al., 2018).

The effect of rhizobium bacteria on the nutritional status of *Medicago* species, and their interaction with a variety of non-nodulating PGPR, has been described throughout the years, with beneficial bacteria being isolated from different plant tissues (Brereton et al., 2020; Cedeño-García et al., 2018; Grover et al., 2021; Kisiel and Kępczyńska, 2016; Liu et al., 2014; Rosier et al., 2021; Zhu et al., 2020). A summary of PGPR interactions with *Medicago* legumes is listed in **Table 1**. Direct and/or indirect mechanisms can mediate the general effects of this kind of bacteria on plants: the production of organic volatile compounds (Ledger et al., 2016), nutrient solubilization (Orellana et al., 2022), rhizosphere acidification by H^+^ liberation (Grover et al., 2021), increased nutrient cycling, enzymatic activity (Grover et al., 2021), secretion of phytohormones such as IAA, cytokinins, gibberellins, and abscisic acid production; and/or the modulation of plant hormone-related pathways (Poupin et al., 2016). Given their influence on plant development, PGPR selection has proven effective in enhancing plant tolerance to abiotic stress (Grover et al., 2021; Marín et al., 2021) and to metal stress (Asad et al., 2019). Individual strains or co-inoculation of metal-tolerant PGPR have shown plant growth effects, enhanced root and shoot fresh and dry weight, as well as shoot height or root length under metal exposure (Flores-Duarte et al., 2022b; Jian et al., 2019; Ju et al., 2019; Li et al., 2017, 2021; Raklami et al., 2019) **Table 1**. Accordingly, inoculating *M. sativa* with beneficial rhizobacteria producing phytohormones and/or siderophores enhances root and shoot growth under Al (Tamariz-Angeles et al., 2021), Pb (Fang et al., 2020), Cu, and Zn stress (Jian et al., 2019), as well as under Cd exposure (Sepehri and Khatabi, 2021), evidencing the microbial role in metal toxicity tolerance.

**Table 1:** Representative PGPR-associated effects on *Medicago* species growth and development under normal or abiotic stress conditions.

Effect	PGPR	Condition	References
Total fresh or dry weight.	*Pantoea* sp. Y4-4	Cu, Zn contaminated soil.	(Li et al., 2017)
	*Bacillus subtilis* subsp. *subtilis* NRBC002. *B. subtilis* NRBC003*, B. megaterium* ATCC14581	Normal, NaCl-stress.	(Zhu et al., 2020)
	*B. niacini* KK 1b*, B. megaterium* KK 11*, Lysinibacillus fusiformis* KK 2, KK 3*, Paenibacillus borealis* KK 4*, P. amyloliticus* KK 9a*, P. odorifer* KK 1a, *S. meliloti* KK 13*, Stenotrophomonas maltophilia* KK 8b, KK 9b, *Citrobacter murliniae* KK 10*, Leclercia adecarboxylata* KK 6*, Raoultella planticola* KK 8a, *Pseudomonas brassicacearum* KK 5*, P. corrugata* KK 7, KK 12.	Normal	(Kisiel and Kępczyńska, 2016)
	*S. meliloti* CCNWSX0020	Normal, Cu-stress, Zn-stress, Cu/Zn contaminated soil.	(Duan et al., 2019; Jian et al., 2019)
Shoot fresh or dry weight.	*S. meliloti* Sm78D, 108	Normal, NaCl-stress.	(Baha and Bekki, 2015)
	*Ensifer medicae* MA11	Normal. Nutrient deficient soil. As, Cd, Cu, Zn contaminated soil.	(Flores-Duarte et al., 2022b)
	*Pantoea* sp. Y4-4	Cu, Zn contaminated soil.	(Li et al., 2017)
	Same as for total frsh or dry weight	Normal	(Kisiel and Kępczyńska, 2016)
	*B. filamentosus* YSP110	Pb-stress	(Yahaghi et al., 2019)
	*B. cereus* YSP69	Zn-stress	(Yahaghi et al., 2019)
	*B. subtilis* TTL1	Cd contaminated soil.	(Li et al., 2021)
	*Agrobacterium tumefaciens* CCNWGS0286	Normal, Cu-stress, Zn-stress	(Jian et al., 2019)
	*Pseudomonas* sp. NT27	Cr-stress	(Tirry et al., 2021)
	*P. aeruginosa* LJL-13*, E. aerogenes* LJL-5	NaCl/Alkaline soil	(Liu et al., 2019)
	*Variovorax gossypii* JM-310, *V. paradoxus* S110	Nutrient deficient soil.	(Flores-Duarte et al., 2022b)
	*S. meliloti* CCNWSX0020	Normal, Cu/Zn and Pb/Cd contaminated soil.	(Duan et al., 2019; Jian et al., 2019)
Root fresh or dry weight.	*S. meliloti* Sm78D, 108	Normal	(Baha and Bekki, 2015)
	*E. medicae* MA11	Normal. Nutrient deficient soil. As, Cd, Cu, Zn contaminated soil.	(Flores-Duarte et al., 2022a)
	*B. licheniformis* YSP149*, B. filamentosus* YSP110	Normal, Pb-stress.	(Yahaghi et al., 2019)
	*Paenibacillus illinoisensis* YSP24	Pb-stress.	(Yahaghi et al., 2019)
	*P. azotoformans* YSP130*, B. cereus* YSP4	Normal, Pb-stress, Zn-stress.	(Yahaghi et al., 2019)
	Same as for total frsh or dry weight	Normal	(Kisiel and Kępczyńska, 2016)
	*A. tumefaciens* CCNWGS0286	Normal, Cu/Zn contaminated soil.	(Jian et al., 2019)
	*P. aeruginosa* LJL-13*, E. aerogenes* LJL-5	NaCl/Alkaline soil.	(Liu et al., 2019)
	*B. subtilis* TTL1	Cd contaminated soil.	(Li et al., 2021)
	*Pseudomonas* sp. NT27	Cr-stress.	(Tirry et al., 2021)
	*V. gossypii* JM-310, *V. paradoxus* S110	Nutrient deficient soil.	(Flores-Duarte et al., 2022b)
	*B. subtilis* subsp. *subtilis* NRBC002 *B. subtilis* NRBC003	NaCl-stress.	(Zhu et al., 2020)
	*B. megaterium* ATCC14581	Normal	(Zhu et al., 2020)
	*Pantoea* sp. Y4-4	Cu-stress	(Li et al., 2017)
	*B. cereus* YSP4	Normal, Pb-stress, Zn-stress.	(Yahaghi et al., 2019)
	*B. subtilis* NRBC003	NaCl stress.	(Zhu et al., 2020)
	*Brevibacterium frigoritolerans* YSP40; *P. illinoisensis* YSP24. *P. azotoformans* YSP130	Normal, Pb-stress.	(Yahaghi et al., 2019)
	*S. meliloti* B399, *Bacillus* M7c, *Pseudomonas* FM7d	Normal	(Guiñazú et al., 2010)
Root surface area and/or volume.	*B. subtilis* subsp. *subtilis* NRBC002	Normal, NaCl-stress.	(Zhu et al., 2020)
	*B. subtilis* NRBC003	NaCl-stress.	(Zhu et al., 2020)
	*B. filamentosus* HL6, B. *subtilis subsp. stercoris* HG12	NaCl-stress	(Ma et al., 2024)
Seed germination percentage and/or rate.	*Pseudomonas mucoides* N4 and N8*, E. meliloti* N10 and N12, and pair combinations.	As, Cd, Cu, and Zn	(Flores-Duarte et al., 2022a)
	*V. gossypii* JM-310, *V. paradoxus* S110, *Ensifer medicae* MA11, and pair combinations.	As, Cd, Cu, and Zn.	(Flores-Duarte et al., 2022b)
	*B. muralis* GN-2, *B. niacini* GN-8, *Pseudomonas* FM7d	Normal.	(Cedeño-García et al., 2018)
Symbiotic interactions (Nodule and weight, nitrogenase activity).	*A. tumefaciens* CCNWGS0286	Cu-stress, Zn-stress, Cu/Zn stress	(Jian et al., 2019)
	*V. gossypii* JM-310, *V. paradoxus* S110	Normal. Nutrient deficient soil. As, Cd, Cu, Zn contaminated soil.	(Flores-Duarte et al., 2022b)

Numerous studies have demonstrated that diverse microorganisms from different *Medicago* species and their rhizospheres exhibit characteristics and potential to be PGPR. IAA-producing bacterial strains, like *P. fluorescens* UM270 and *E. cloacae* MSR1, have been isolated from *M. truncatula* and *M. sativa* rhizosphere and nodules, respectively, and characterized by their beneficial effects on plant growth (Khalifa et al., 2016). Many *Bacillota* species, including *Bacillus*, *Lysinibacillus*, and *Paenibacillus*, were also isolated from *M. sativa* rhizosphere and characterized as good phytostimulators of *M. truncatula* growth (Kisiel and Kępczyńska, 2016). Additionally, Brereton et al. (2020) reported localization of potential PGPR, *Neorhizobium huatlense, Nocardioides luteus*, and *P. nitroguajacolicus* in the *M. sativa* rhizosphere under oil-contaminated soils. More significantly, IAA-producing *Bacillus filamentous* YSP110 and *B. cereus* YSP4 strains were also isolated from alfalfa to explore their effects on shoot growth and metal accumulation (Yahaghi et al., 2019), revealing that the two species increased both plant growth and metal accumulation in roots when plants were exposed to Zn and Pb. However, it is essential to note that various microorganisms with PGPR characteristics able to enhance the metal tolerance of *M. sativa* have been isolated from diverse environments. These include rhizospheric soil from Cu mine tailings (Liu et al., 2014) and the Tinto River estuary (Mesa et al., 2015), as well as in contaminated soil with heavy metals (Tamariz-Angeles et al., 2021).

Co-inoculation with PGPR has demonstrated synergistic effects on the growth of *Medicago* species. For example, the co-inoculating *Agrobacterium tumefaciens*, an IAA producer, and *Sinorhizobium meliloti*, which promotes nodulation, enhanced the development of *M. lupulina* under Cu and Zn stress (Jian et al., 2019). **Table 1**. In another study, different combinations of the bacteria, *Variovorax gossypii, V. paradoxus*, IAA producers, and *E. medicae* as nodulating strains, showed improved *M. sativa* growth, including enhanced nodulation, nitrogen content in shoots, and enhanced metal stress response after the inoculation of the three bacterial strains (Flores-Duarte et al., 2022a). However, Liu et al. (2014) reported decreased shoot height and improved above- and below-ground biomass of *M. sativa* due to mixed co-inoculation of PGPR with ACC deaminase activity and free-living N-fixing bacteria under exposure to Cu mining tailings, compared with co-inoculation with multiple N-fixing bacteria alone. Rosier et al. (2021) showed that co-culture of the PGPR *B. subtilis* UD1022 with *S. meliloti* Rm8530 affected the growth of *M. truncatula*. The authors attributed this effect to an indirect interaction between bacteria that reduced *S. meliloti* Rm8530 biofilm formation and downregulated quorum-sensing genes responsible for symbiotically active biofilm formation, which is essential for nodule initiation on the roots.

PGPR can contribute to ion-exclusion mechanisms by releasing metal-chelating compounds, such as organic acids, siderophores, and biosurfactants, thereby enhancing metal solubilization and mobility in the rhizosphere and leading to metal leaching from the root zone (Asad et al., 2019). Farh et al. (2017) observed that inoculation of PGPR, specifically *Pseudomonas simiae* N3 and *Burkholderia ginsengiterrae* N11-2, enhanced the expression of Al^3+^ tolerance genes in *A. thaliana.* This led to increased levels of the Al-activated malate transporter *AtALM1* and the Al-induced protein *AtAIP*, resulting in lower root Al^3+^ accumulation, linked to external tolerance mechanisms. **Figure 1**, inset. Similarly, Khanna et al. (2019) reported that *Solanum l*ycopersicon, when subjected to Cd stress, enhanced the expression of metal transporters in the presence of *P. putida* and *B. gladioli*, which promote plant growth. Regarding *Medicago*-reported interactions, microbial inoculants improving Cu (Liu et al., 2014), Cd (Sepehri and Khatabi, 2021), and Pb (Tamariz-Angeles et al., 2021) tolerance in alfalfa were all capable of producing siderophore chelators for Al or Fe.

Metallic contamination reduces nutrient availability, making metal tolerance a critical factor in nutrient acquisition. Consequently, nodulating strains of *Sinorhizobium or Ensifer* have also been used as inoculants for *Medicago* species due to their effective N-fixation capabilities (Baha and Bekki, 2015; Flores-Duarte et al., 2022b). For example, the root and shoot N and P contents of *M. sativa* have been reported to increase in response to a potential PGPR/Cu-resistant *S. meliloti* strain under metal exposure (Duan et al., 2019). Similarly, non-nodulating bacteria, such as *Stenotrophomonas* sp., *Pseudomonas* sp., *Agrobacterium* sp., *Ochrobactrum* sp., *Advenella incenata*, and *Acinetobacter calcoaceticus* with high N fixation and organic and inorganic phosphorus solubilization, have also been selected and effectively applied as bioinoculants to promote the growth of *M. sativa* under both normal and metal-stress conditions (Liu et al., 2014). Moreover, several *Bacillus* spp. and *Pseudomonas* spp. strains with inorganic P, K, and Zn solubilization capabilities have been evidenced to promote the growth and nutrient acquisition of *M. sativa* co-inoculated with *S. meliloti* (Cedeño-García et al., 2018; Guiñazú et al., 2010; Kisiel and Kępczyńska, 2016; Liu et al., 2014; Tirry et al., 2021; Zhu et al., 2020). **Table 1**.

Furthermore, inoculation of metal-solubilizing PGPR has been proposed as a strategy to improve the uptake of various ionic metals, such as Cd, Cu, Zn, and Pb in plants, thereby enhancing growth and phytoextraction efficiency (Li et al., 2021; Yahaghi et al., 2019). **Table 1**. Alfalfa has been regarded as a valuable legume model for phytoextraction applications due to its perennial, fast-growing, highly dense root system, biomass-forming capabilities, and strong overall temperature tolerance (Liu et al., 2019). Alfalfa inoculation with *B. cereus* YSP4 and *B. filamentous* YSP110 enhanced Zn and Pb tissue concentrations (Yahaghi et al., 2019). In addition, Flores-Duarte et al. (2022b) observed increased Cd^2+^, Cu^2+,^ and Zn^2+^ accumulation in *M. sativa* roots and shoots due to *E. medicae* MA11 individual inoculation and co-inoculation with the IAA and ACC deaminase-producing strains *V. gossypii* JM-310 and *V. paradoxus* S110. In other cases, plant growth promotion, mainly of the shoot and root biomass, increased the content of Cu, Cr, Pb, and Zn in both plant structures, therefore showing promising potential for metal removal of contaminated soils (Jian et al., 2019; Li et al., 2017; Tirry et al., 2021). Thus, although numerous studies have shown that PGPR can enhance Medicago tolerance to metal stress, key gaps remain. Most findings stem from controlled conditions, with limited understanding of how microbial consortia perform in field soils. The molecular coordination between plant responses and PGPR activity under multimetal stress is also poorly resolved. Future research should focus on genotype–strain specificity and integrative omics to design effective, context-adapted bioinoculants for remediation and sustainable legume cultivation.

### Interactions among *Medicago*-AMF-metals

AMF inoculation in *Medicago* species promotes plant growth and nutrient acquisition (Dahlia et al., 2025) and is particularly beneficial under low−fertility or metal−stress conditions. These interactions are associated with enhanced metal tolerance through both internal and external detoxification mechanisms (Lin et al., 2007; Peng et al., 2020; Redon et al., 2009). The effect of AMF on alfalfa plants has been extensively studied. Inoculation with *Glomus mossae* increased the number of nodules in alfalfa growing in soil contaminated with Cu, Zn, Pb, and Cd (Lin et al., 2007). Similarly, *G. intraradices* also enhanced nodule formation under Cd, Zn, and Pb stress (Redon et al., 2009). Additionally, Cd-immobilization functions have been reported in dark septate endophytes, a subgroup of Ascomycetes that interact closely with alfalfa (Hou et al., 2020). The association between M. sativa and the AMF *Rhizophagus irregularis* induced phytochelatin production under Cd stress in greenhouse experiments. Expression of the phytochelatin synthase gene MsPCS1 was significantly induced in cells of inoculated plants, suggesting that chelator-mediated detoxification is active and that Cd is translocated into vacuoles in root tissues. Accordingly, fungal inoculation significantly reduced metal accumulation in shoots and promoted aboveground biomass growth (Motaharpoor et al., 2019). Phytochelatin production, MsPCS gene expression, and metal-chelating GSH content were also increased in the roots of G. mossae-inoculated alfalfa plants exposed to Cd in combination with a 3°C increase in temperature (Gao et al., 2023). The lower phytochelatin accumulation and MsPCS response in shoots are presumed to be due to lower Cd levels in aerial tissues relative to uninoculated controls, consistent with a role for the fungus in preventing metal transfer.

Co-inoculation of AMF and a community of rhizosphere bacteria reduced Cd-induced lipid peroxidation and ROS stress in alfalfa by increasing the activity of antioxidant enzymes (Wang et al., 2021). Additionally, AMF interactions have been shown to influence the antioxidant nitrosative stress response in *M. truncatula* roots. This response involves excessive NO accumulation in plant tissues under metal stress, which can alter protein conformation (Sujkowska-Rybkowska et al., 2018) and even impair root growth and elongation (**Figure 1**) (Chen et al., 2014). NO production has been shown to contribute to root growth inhibition (Das et al., 2026; Gupta et al., 2024).

Under metal stress, fungal interactions have indeed shown positive effects in promoting *Medicago* legume nutrient uptake, mainly N and P, suggested to be an effect of enhanced symbiotic interactions with N-fixing rhizobia, better root architecture development, and improved nutrient availability in the rhizosphere (Hou et al., 2020; Lin et al., 2007). Peng et al. (2020) demonstrated a significant effect of inoculation and P treatment in *M. sativa* roots and shoots due to *G. mossae* BGC YNo2 in Se-contaminated groups, but not with the sole fungal inoculation. *G. mossae* species and *G. intraradecis*, respectively, enhanced *M. sativa* and *M. truncatula* shoot and root dry weight under excessive multi-metal exposure (Cd, Cu, Pb, Zn) while increasing metal uptake of Cd, Cu, and Zn in roots and shoots (Lin et al., 2007; Redon et al., 2009). Similarly, dark septate endophytes *Acrolymma vagum* and *Scytalidium lignicola*, isolated from metal-contaminated areas, promoted root and shoot biomass of *M. sativa*, respectively, improving root length, architecture, and Cd-accumulation, and enhancing tolerance to excessive Cd exposure (Hou et al., 2020). In addition, *M. sativa* and *M. truncatula* root phytostabilization of Cu, Cd, Se, and Zn has been shown to increase with inoculation of *G. mosseae* and *G. intraradecis*, linked to reduced translocation factors and healthy shoot growth (Lin et al., 2007; Redon et al., 2009). In the context of tripartite symbiotic relationships with nodulating rhizobacteria, AMF inoculation of various *Glomus* species in *M. sativa* and *M. truncatula* showed increased nodulation under multi-metal exposure (Lin et al., 2007; Redon et al., 2009). Redon et al. (2009) associated the significant increase in biomass of *G. intraradecis no. 1*-inoculated *M. truncatula* plants with the more substantial presence of root nodules, which could explain the increases in N content and the shoot growth-promoting effects, rather than being a consequence of fungal inoculation alone. Symbiotic fungal associations with *Medicago* add a layer of complexity to the plant’s response to metal stress, influencing growth, nutrient acquisition, interactions with rhizosphere bacteria, and both metal uptake and tolerance mechanisms. Understanding these multifaceted interactions is essential to fully characterize how Medicago adapts to contaminated environments.

## Conclusions and future perspectives

This review highlights that *Medicago* species deploy a wide range of coordinated physiological, biochemical, and microbial strategies to withstand metal stress. These include organic acid exudation, modulation of phytohormone signaling—especially auxin and ethylene pathways—and activation of antioxidant defenses. Such mechanisms, which alter root growth patterns and reduce metal accumulation, have been consistently documented in *M. sativa*, *M. truncatula*, and related taxa, though substantial variation remains between tolerant and sensitive genotypes.

Beyond intrinsic plant adaptations, rhizospheric microorganisms—including plant growth-promoting rhizobacteria (PGPR) and arbuscular mycorrhizal fungi (AMF)—play essential roles in enhancing *Medicago* tolerance. PGPR contribute through nutrient solubilization, phytohormone and siderophore production, and modulation of stress-related signaling, while AMF improve nutrient acquisition, metal sequestration, and antioxidant balance. Co-inoculation strategies often amplify these effects, though results differ widely depending on microbial composition, plant genotype, and soil conditions.

Importantly, many of these mechanisms parallel those observed in other plant systems. For example, Arabidopsis, rice, sorghum, barley, and soybean exhibit similar strategies, such as MATE-mediated citrate efflux, ALMT-dependent malate release, and hormone-driven root growth modulation under metal stress (Jin et al., 2024; Wang et al., 2023). Understanding how these diverse systems converge or diverge offers valuable context for interpreting *Medicago* biology and identifying traits with strong translational potential for crop improvement and phytoremediation.

However, despite substantial progress, major knowledge gaps persist. For instance, aluminum studies are less common than other metals, especially for PGPR and AMF. On the other hand, much of the available data comes from controlled laboratory experiments using single microbial strains or single-metal exposures, which do not reflect the ecological complexity of metal-contaminated soils. The specificity and stability of genotype–microbe associations, the plasticity of microbiome assembly under stress, and the molecular integration of microbial and plant responses remain poorly resolved. Additionally, it remains unclear how *Medicago*-derived mechanisms compare functionally with those in other legumes and non-legume models under multimetal or fluctuating environmental conditions.

Future research should therefore adopt integrative and comparative approaches. Multi-;omics tools—metagenomics, transcriptomics, metabolomics, and ionomics—combined with ecological modeling and high-resolution imaging, can help resolve how plant–microbe–metal interactions operate *in situ*. Comparative studies across legume and non-legume models will clarify which tolerance strategies are universal and which are lineage-specific. Field-based testing, synthetic microbial consortia, and genotype-;by-;environment-;by-;microbiome experiments will be crucial for identifying robust, scalable solutions.
